# Genetic Architecture of Anther Extrusion in Spring and Winter Wheat

**DOI:** 10.3389/fpls.2017.00754

**Published:** 2017-05-16

**Authors:** Quddoos H. Muqaddasi, Jonathan Brassac, Andreas Börner, Klaus Pillen, Marion S. Röder

**Affiliations:** ^1^Leibniz Institute of Plant Genetics and Crop Plant ResearchGatersleben, Germany; ^2^Institute of Agricultural and Nutritional Sciences, Martin Luther University of Halle-WittenbergHalle, Germany

**Keywords:** hybrid wheat, anther extrusion, marker trait associations, QTL, linkage disequilibrium, *Triticum aestivum* L.

## Abstract

Hybrid wheat breeding is gaining prominence worldwide because it ensures higher and more static yield than conventionally bred varieties. The cleistogamous floral architecture of wheat (*Triticum aestivum* L.) impedes anthers inside the floret, making it largely an inbreeder. For hybrid seed production, high anther extrusion is needed to promote cross pollination and to ensure a high level of pollen availability for the seed plant. This study, therefore, aimed at the genetic dissection of anther extrusion (AE) in panels of spring (SP), and winter wheat (WP) accessions by genome wide association studies (GWAS). We performed GWAS to identify the SNP markers potentially linked with AE in each panel separately. Phenotypic data were collected for 3 years for each panel. The average levels of Pearson's correlation (*r*) among all years and their best linear unbiased estimates (BLUEs) within both panels were high (*r*(SP) = 0.75, *P* < 0.0001;*r*(WP) = 0.72, *P* < 0.0001). Genotypic data (with minimum of 0.05 minor allele frequency applied) included 12,066 and 12,191 SNP markers for SP and WP, respectively. Both genotypes and environment influenced the magnitude of AE. In total, 23 significant (|log_10_(*P*)| > 3.0) marker trait associations (MTAs) were detected (SP = 11; WP = 12). Anther extrusion behaved as a complex trait with significant markers having either favorable or unfavorable additive effects and imparting minor to moderate levels of phenotypic variance (*R*^2^ *(SP) = 9.75*−14.24%; *R*^2^ (*WP*) = 9.44−16.98%). All mapped significant markers as well as the markers within their significant linkage disequilibrium (*r*^2^ ≥ 0.30) regions were blasted against wheat genome assembly (IWGSC1+popseq) to find the corresponding genes and their high confidence descriptions were retrieved. These genes and their orthologs in *Hordeum vulgare, Brachypodium distachyon, Oryza sativa*, and *Sorghum bicolor* revealed syntenic genomic regions potentially involved in flowering-related traits. Moreover, the expression data of these genes suggested potential candidates for AE. Our results suggest that the use of significant markers can help to introduce AE in high yielding varieties to increase cross fertilization rates and improve hybrid-seed production in wheat.

## Introduction

In many crops, hybrids have almost completely replaced the conventionally bred varieties because of their potential of higher and more stable yields even in marginal environments (Schnable and Springer, 2013). Heterosis in F1 hybrids ensures greater economic yield and static yield stability (Longin et al., 2012; Muhleisen et al., 2014; Zhao et al., 2015). Although, hybrid wheat breeding has a long history, still the wheat hybrid market is very small. This slow progress in hybrid wheat seed production is due to the hermaphroditic cleistogamous architecture of wheat florets which impedes proper anther extrusion to allow sufficient pollen shedding outside the floret. To guarantee a sound hybrid wheat seed production program, the cleistogamous nature of wheat needs an alteration to open flowering (Whitford et al., 2013). This floral modification will allow anthers to extrude during anthesis for sufficient pollen shedding outside the florets, so they may become available for the female lines via wind pollination. Moreover, this floral adjustment will not only profit male lines but female lines as well, since they ought to have open flowers to receive sufficient amount of pollen with receptive stigmas. Subsequently, the existing insignificant cross fertilization rates in wheat can be increased by ensuring a higher level of anther extrusion for appropriate pollen shedding outside of florets.

Male sterility is the most efficient way to ensure the cross pollination in inbreeding crops and this can be achieved in wheat by several means, e.g., cytoplasmic male sterility, genic male sterility and the use of chemical hybridization agents (CHAs) (Perez-Prat and van Lookeren Campagne, 2002; Chen and Liu, 2014; Kempe et al., 2014). The absence of an efficient cytoplasmic male sterility system and toxic effects coupled with CHAs are major reasons for less hybrid wheat seed production (Longin et al., 2012; Whitford et al., 2013). However, currently, the most widely used industrial scale hybrid wheat production system is the induction of male sterility by means of CHAs (Longin et al., 2012). CHAs, applied exclusively on female-parents, render the plants male-sterile. These male-sterile lines (females) receive pollen from neighboring male lines. Nevertheless, besides achieving an efficient male-sterility system and the determination of heterotic groups (Zhao et al., 2015), successful development of hybrid wheat varieties depends upon the modification of floral architecture to ensure high cross-pollination capacity in wheat (Whitford et al., 2013). This can be guaranteed by adequate volume of anther extrusion and viable pollen-shed outside the florets.

Anther extrusion is a complex trait controlled by many genes. The genetic basis of AE has mostly been unveiled via conventional mapping studies (Skinnes et al., 2010; Lu et al., 2013; Buerstmayr and Buerstmayr, 2015). Association mapping (AM) is a more recent method to determine the quantitative trait loci (QTLs) in a non-related germplasm by marker-trait associations (Risch and Merikangas, 1996; Zhu et al., 2008). This method is advantageous over the conventional QTL mapping technique because it profits from the strength of linkage disequilibrium (LD) and higher recombination frequencies available in diverse germplasm and offers an increased mapping resolution, a smaller investment in research time and cost, and an opportunity to survey a wider range of alleles at any given locus (Zhu et al., 2008; Hamblin et al., 2011). Recently, AM studies to unveil the genetic architecture of AE in spring wheat by using mainly DArT and GBS markers (Muqaddasi et al., 2016, 2017) and winter wheat (Boeven et al., 2016) by SNP markers, also suggested a highly quantitative nature of AE.

In this investigation, genome wide association studies were carried out to identify the genetic basis of AE in diverse panels of spring and winter wheat accessions. The 15K SNP marker platform was chosen for its high density and highly informative and genome-wide polymorphic loci—a perquisite for efficient AM studies.

## Materials and methods

### Plant material and phenotyping of anther extrusion

The spring wheat panel consisting of 111 accessions from the Genebank of IPK in Gatersleben (http://www.ipk-gatersleben.de/en/genebank/) was described in Muqaddasi et al. (2016). The winter wheat panel of 96 accessions was previously described in Neumann et al. (2011). The whole set was field-grown in Gatersleben, Germany (51°49′N 11°16′E, 112 m asl) during 3 years (*SP*; 2013, 2014, 2015:*WP*; 2013, 2015, 2016), arranging the accessions as a randomized complete block design (four replicates in 2013 and 2014, and one in 2015 and 2016). The individual plot size was 1 × 1.5 m, and each plot was split into four rows spaced 0.20 m apart. Standard agronomic wheat management practices were applied.

Anther retention (AR; number of non-extruded anthers) was scored on field in both panels 5–10 days post-anthesis for the years 2013, 2014, and 2015 by observing the anthers retained inside the four pairs of primary and secondary florets sampled from the central portion of four spikes per plot. For years 2015 and 2016, AR was scored by taking 10 spikes per plot, harvested 5–10 days post-anthesis and held at −20°C until analysis in the laboratory. Field and laboratory based AR data (2015) were compared to check the usefulness of both methods. Anther extrusion was calculated by subtracting the number of retained (non-extruded) anthers from 24 (since the total number of anthers housed by eight florets is 24) as described in Muqaddasi et al. (2016).

### Statistical analysis

The mean AE in each growing year was used to calculate the best linear unbiased estimates (BLUEs), assuming fixed genotypic effects. The calculations were performed using GenStat *v*16 software (VSN International, Hemel Hempstead, Hertfordshire, UK) applying the “Mixed models REML” module and the “Linear mixed models” function. A Pearson product moment correlation among the growing years and BLUEs was also calculated. The repeatability among the replicates (*R*) and heritability (*H*^2^) among the years were calculated from the individual variance components as $R=\frac{\sigma_{G}^{2}}{\sigma_{G}^{2}+\left(\frac{\sigma_{e}^{2}}{nR}\right)}$ and $H^{2}=\frac{\sigma_{G}^{2}}{\sigma_{G}^{2}+\left(\frac{\sigma_{G\times E}^{2}}{nE}\right)+\left(\frac{\sigma_{e}^{2}}{nE\times nR}\right)}$ where, $\sigma_{G}^{2}$ represents the variance of genotype, $\sigma_{G\times E}^{2}$ is the variance of the interaction of genotype by environment, $\sigma_{e}^{2}$ the variance of error, *nE* is the number of environments and *nR* the number of replicates (Nyquist and Baker, 1991).

### Genotyping, population structure, and kinship estimates

All 207 wheat genotypes were analyzed using a 15K Infinium SNP array, which is an optimized and reduced version of the 90K iSELECT SNP-chip described by Wang et al. (2014). The development of the 15K SNP-chip and genotyping was performed by TraitGenetics GmbH (http://www.traitgenetics.com). The initial number of polymorphic loci was 13,006 for both panels. The SNP-chip data is available at digital object identifier (DOI) http://dx.doi.org/10.5447/IPK/2017/4. This DOI was created with e!DAL (Arend et al., 2014). A minor allele frequency threshold (MAF) of 5% was applied prior to the determination of marker-AE associations which reduced the size of the data matrices to 12, 066 × 111 for SP and 12, 191 × 96 for WP.

Genetic relationships among the SP and WP accessions were investigated graphically via principal component analysis (PCA) based on SNP genotypes in software R (Team, 2016). The first two principal components of each panel were depicted in two-dimensional space to show the clustering of accessions.

Assuming that the accession share some degree of relatedness, a genetic variance-covariance kinship matrix (*K*) as coefficient of co-ancestry (Lynch and Walsh, 1998) was derived from all SNP markers (*MAF* > 5%) between all pairs of accessions in each panel individually by using GenStat *v*16.

### Marker trait association and linkage disequilibrium assessment

Genome-wide association scan was performed by following Yu et al. (2006). Marker trait associations (MTAs) involving AE and the SNP markers were identified using the “QTL analysis” module and the “Single trait association analysis” function implemented in GenStat *v*16, applying a variance-covariance kinship matrix ($VCOV\left(G_{i}\right)=2K\sigma_{g}^{2}$) for the purpose of correction for population stratification (Lynch and Walsh, 1998).

Assuming the genetic relatedness among accessions, the following mixed model for MTA analysis on BLUEs dataset with SNP markers was used:

$$y_{i}=\mu +\alpha x_{i}+G_{i}+(G_{i}+e_{i}),$$

where, *y*_*i*_ is the AE's BLUE value for *i*^*th*^ genotype, μ is the overall intercept, α is the marker effect, *x*_*i*_ is the genetic predictor for *i*^*th*^ genotype, *G*_*i*_ is the effect of *i*^*th*^ genotype and *e*_*i*_ is the error for genotype *i*. The residual effect (*G*_*i*_ + *e*_*i*_) was assumed to arise from a normal distribution as ~*N*(0, σ^2^) which imposed a genetic variance-covariance structure. Genetic relatedness (structure) among accessions was considered by assuming the random genotype effect $G_{i}\sim N\left(0,2K\sigma_{g}^{2}\right)$. The threshold of |*log*_10_(*P*)| > 3.0 was set in GenStat *v*16 for the detection of significant MTAs. The MTA analysis was performed separately for each panel based on BLUEs. Observed vs. expected |*log*_10_(*P*)| value distributions were displayed in the form of quantile-quantile (qq) plots. For the set of markers unlinked to an AE-QTL, the |log_10_(*P*)| values would be expected to be uniformly distributed along the diagonal, however, extremely large deviations are indicative of spurious associations (Yu et al., 2006; Kang et al., 2008). The additive contribution of each linked marker was calculated with GenStat *v*16: negative effects reflect a decrease in AE (unfavorable alleles), and positive ones an increase (favorable alleles). For the bi-allelic SNP markers the effects were related to the respective allele with minor allele frequency at each locus. TASSEL *v*3.0 (Bradbury et al., 2007) was used to calculate the phenotypic variances (*R*^2^) explained by individual markers.

By using a set of mapped SNP markers (SP = 10,578; WP = 10,649) genome-wide linkage disequilibrium (LD) was studied in both panels. Intra-chromosomal and genome-wide quantification and graphical representation of LD heat maps and intra-chromosomal LD decay were accomplished with GenStat *v*16 and R (Team, 2016) by plotting the squared correlation coefficients (*r*^2^) vs. map distances in centiMorgans (cM).

### Analysis of significant LD regions and synteny with related grasses

The 15K SNP Infinium array provided genome-wide polymorphic markers in high density. We performed the pairwise marker LD analysis for each chromosome individually for both panels. We set the significant LD regions by assuming significant marker to be in LD with other markers on the same chromosome by setting the threshold of *r*^2^ ≥ 0.30. Thus, we extracted the flanking sequences of all the markers from wheat 90K database (Wang et al., 2014) associated with AE in significant LD regions (*r*^2^ ≥ 0.30) and blasted (BLASTN; Altschul et al., 1990) against the wheat genome assembly (IWGSC1 + popseq) (Consortium, 2014) to find their corresponding genes and transcripts. The functional descriptions of these genes were retrieved from (ftp://ftpmips.helmholtz-muenchen.de/plants/wheat/IWGSC/genePrediction_v2.2/). All the markers sequences were blasted in Geneious *v*9.1.5 (Kearse et al., 2012).

Additionally, the orthologs of the corresponding genes of the close relatives of wheat i.e., *Brachypodium distachyon, Sorghum bicolor*, rice, and barley were retrieved from Wang et al. (2014) and barley sequence assembly RefSeq *v*1.0 (International Barley Sequencing Consortium pre-publication access). Furthermore, the wheat transcript IDs of all the genes in significant LD regions were given in http://www.wheat-expression.com to retrieve their expression profiles in above-ground plant tissues at log2 transcripts per million (tpm) scale (Borrill et al., 2016). Heat maps of expression profiles were created by using the R package gplots (Warnes et al., 2009) in R (Team, 2016). We portioned the expression data profiles of above ground tissues in two groups i.e., flowering and non-flowering tissue types and calculated the mean expression for all transcripts. One way repeated-measures (RM) analysis of variance (ANOVA) was used to determine significant difference between expression profiles of flowering and non-flowering tissue types. Differences in the means were determined by Holm-Sidak *post-hoc* test (Holm, 1979) by setting a statistical significance of *P* < 0.05. Statistical tests were done in SigmaPlot *v*13.0.

## Results

### Phenotypic data analyses

**Figure 1:**
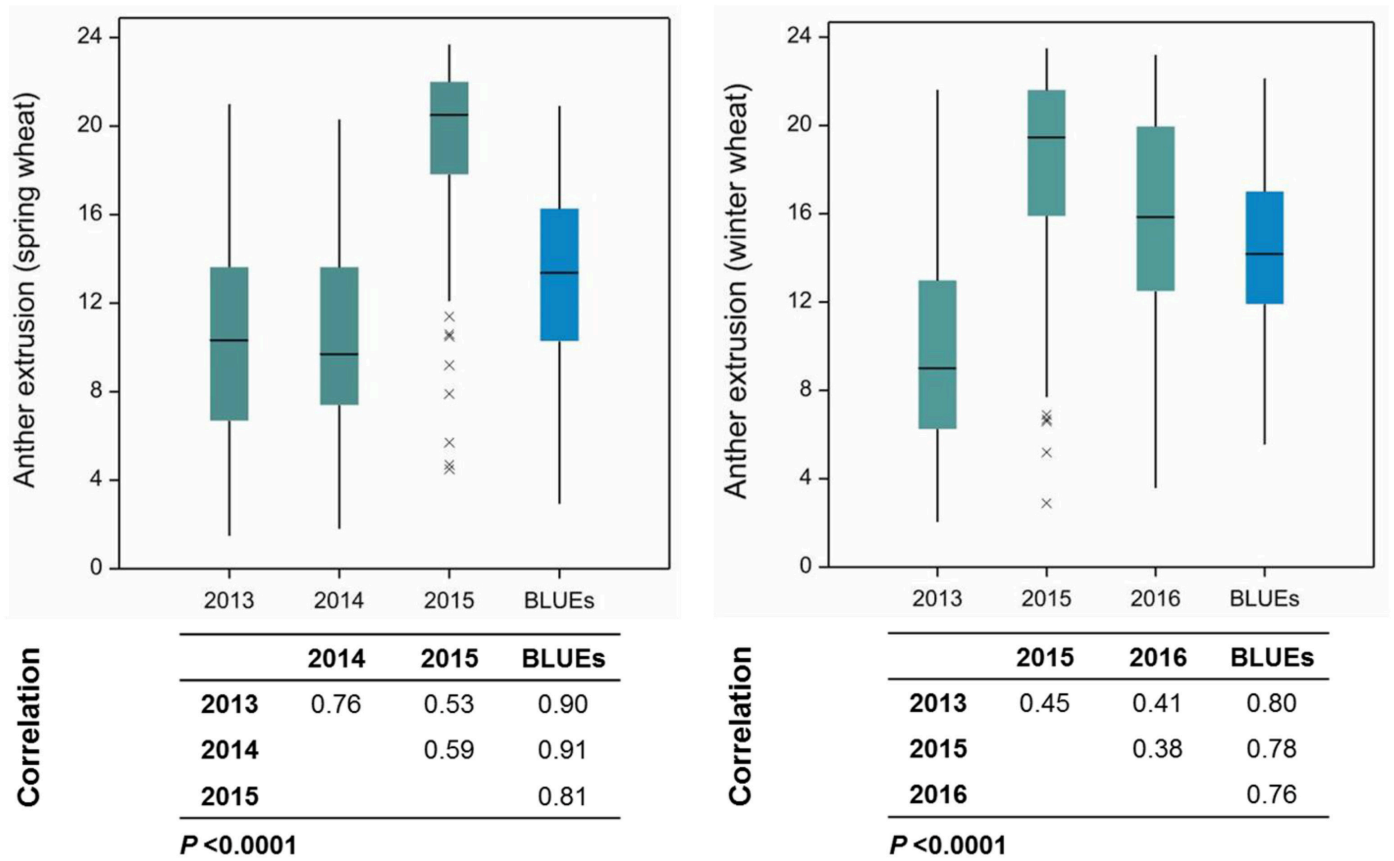
**Boxplots and Pearson's product moment correlations among growing years and their Best Linear Unbiased Estimates (BLUEs) in spring and winter wheat panels**. Numbers on the Y-axis represent the number of extruded anthers. xs indicate the outlier accessions.

**Figure 2:**
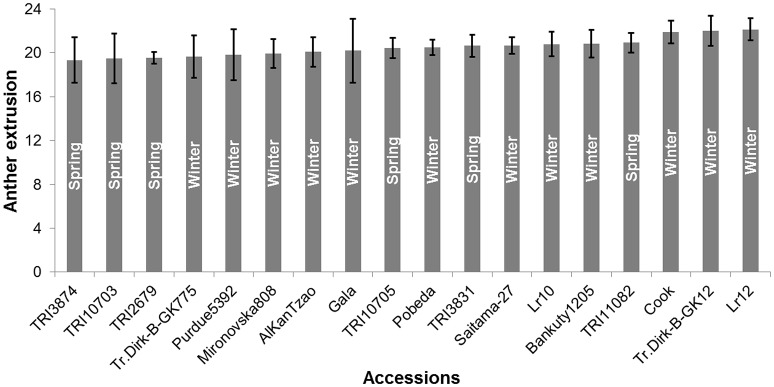
**Anther extrusion (AE) performance of the top anther extruding accessions based on Best Linear Unbiased Estimates (BLUEs) ±SE**. Six spring and 12 winter wheat accessions showed AE >80%. The growth habit of the accessions is indicated within the bars. Numbers on the Y-axis represent the number of extruded anthers.

Anther extrusion (AE) was scored for 207 wheat accessions (111 spring and 96 winter) and showed a wide range of variation among accessions. The habit of growth, country of origin and AE scores of all 207 accessions are given in Table S1. For the year 2015, two methods to measure the AE in both spring (SP) and winter wheat (WP) panels were adopted, i.e., (1) direct scoring of the anthers on the field and (2) collecting and freezing the spikes and determining the AE in the laboratory. Both methods strongly correlated with one another (*Pearson*′*sr*(SP) = 0.81, *P* < 0.0001; *r*(WP) = 0.85, *P* < 0.0001) (Figure S1). The laboratory-based method was preferred for the subsequent genetic analysis, because it avoided difficulties related with the on field AE scoring and lodging of the non-dwarf accessions and because it allowed the processing of large numbers of accessions. The BLUEs of both SP and WP approximated a normal distribution and ranged from 2.94 to 20.92 (mean = 13.24) for SP and from 5.55 to 22.14 (mean = 14.44) for WP (Table S1). The observed range in AE was comparable in the 2013 and 2014 trials, while the 2015 and 2016 experiments produced higher mean values in both panels (Figure 1). The extent of the AE Pearson's correlation among growing years and BLUEs ranged from *r* = +0.53−0.91 in SP and *r* = +0.38−0.80 in WP (Figure 1). The repeatability levels in all years were >0.95 in both panels. The broad sense heritability was 0.84 across years for SP and 0.65 for WP. Six SP and 12 WP accessions showed AE >80% based on their BLUEs ranging from 19.33 to 20.92 (SP) and 19.65 to 22.14 (WP) (Table S1), and the trait was stable across years (Figure 2).

### Marker distribution, linkage disequilibrium, and population structure

The imposition of minor allele frequency (MAF) threshold of 5% reduced the SNP marker set to 12,066 markers for SP and 12,191 for WP, of which 10,578, and 10,649 had known map locations, respectively (Wang et al., 2014), spreading unevenly across all three constituent genomes; however, each of the 21 chromosomes was represented (Figure S2). Almost half of the markers mapped to B-genomes in both panels (SP = 49.42%; WP = 49.33%) followed by A-genomes (SP = 39.08%; WP = 38.87%), while D-genomes had the lowest marker coverage (SP = 11.50%; WP = 11.80%).

Intra-chromosomal and genome-wide linkage disequilibrium (LD) assessment was performed using adjacent (pair-wise) SNP loci which revealed that LD decayed rapidly with increasing genetic map distances. The LD analyses of the chromosomes harboring the markers significantly associated with the AE (Figures S3, S4) showed a considerable variability in chromosome-wise and genome-wide analysis of LD. This suggested that the power of GWAS can be improved by increasing the polymorphic loci density, especially on the D-genome which is least represented among all genomes.

**Figure 3:**
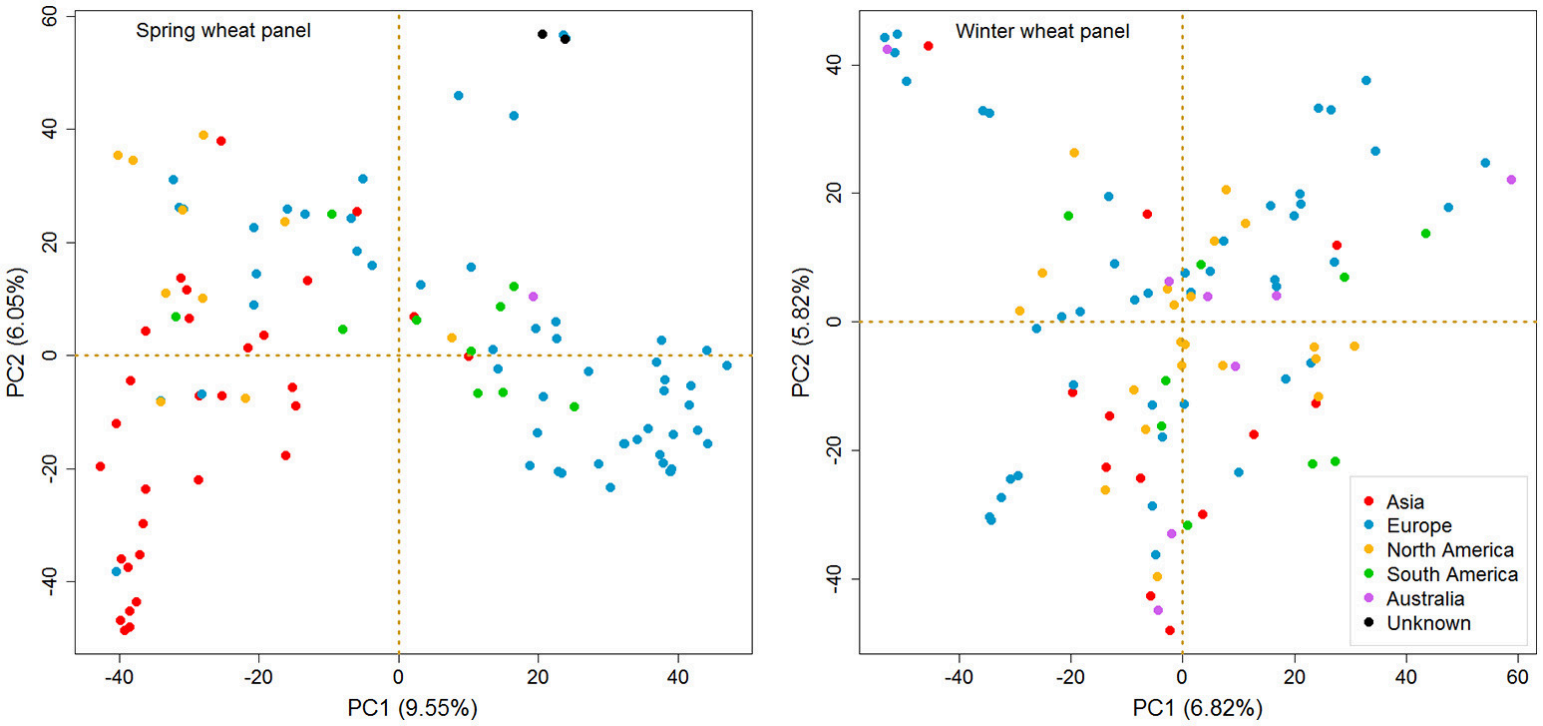
**Principal component analysis (PCA) of spring and winter wheat panels based on SNP genotypes (MAF >5%)**. The spring wheat accessions bred in Europe and Asia form recognizable clusters, but those bred in the Americas do not. There is no pronounced clustering among winter wheat accessions. Color code is given in the figure.

A PCA based test for the existence of clustering among accessions for both panels is illustrated in Figure 3. In order to simplify the PCA plots, we highlighted the accessions with different colors based on their continent of origin. A clear separation was found between the European and Asian accessions in SP, while there was absence of pronounced clustering for the American ones. For WP, no distinct grouping of sub-populations was observed.

### Marker-trait associations for anther extrusion

**Figure 4:**
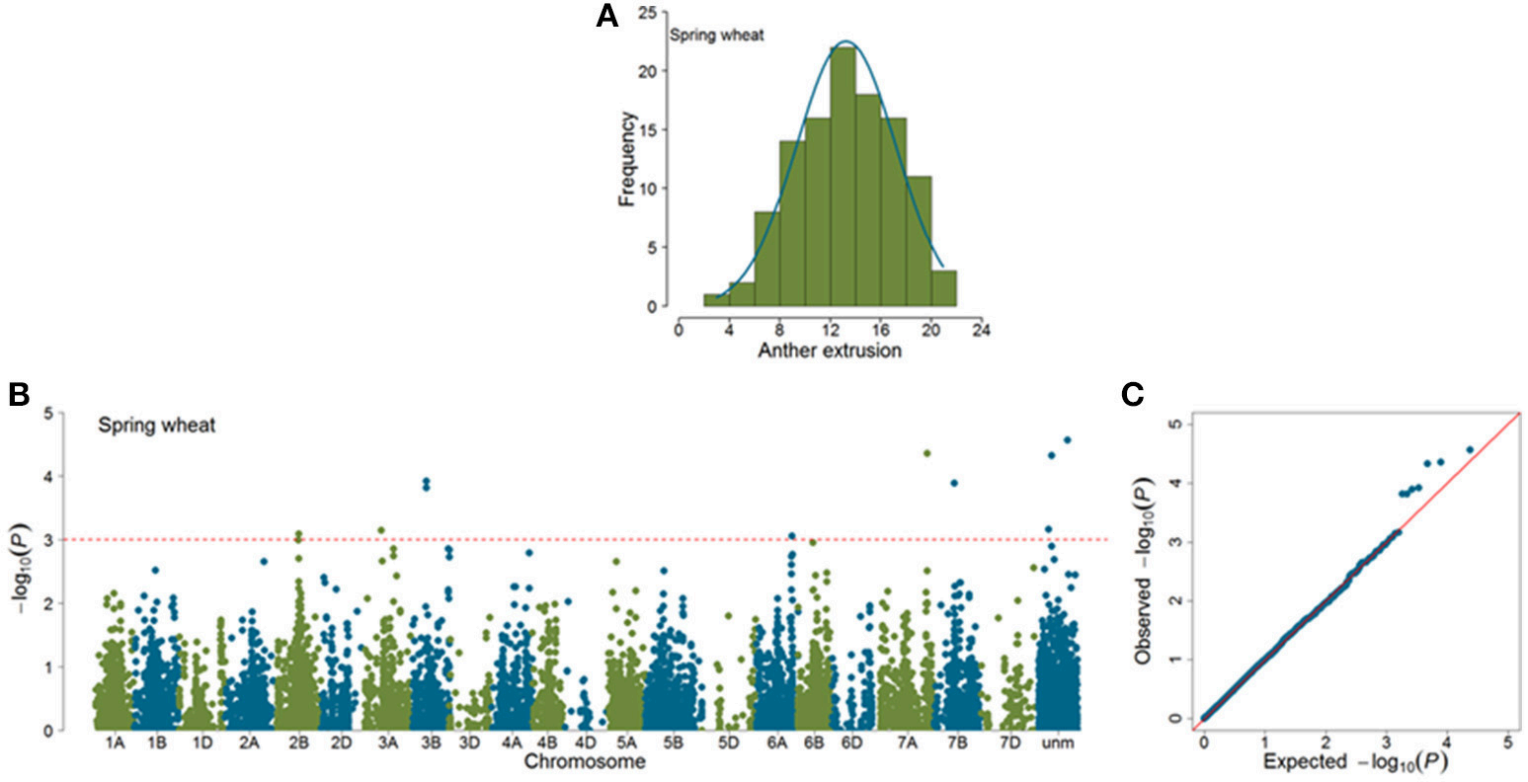
**Summary of genome-wide association studies results of anther extrusion (AE) in spring wheat panel (SP)**. **(A)** Distribution of Best Linear Unbiased Estimates of AE **(B)** Manhattan plots based on mixed linear model using kinship matrix (*K*) to correct for population stratification. Horizontal dashed line corresponds to the threshold (|*log*_10_(*P*)| > 3.0) for MTA estimation. **(C)** Quantile-quantile plots depicting expected vs. observed |log_10_(*P*)| values.

**Figure 5:**
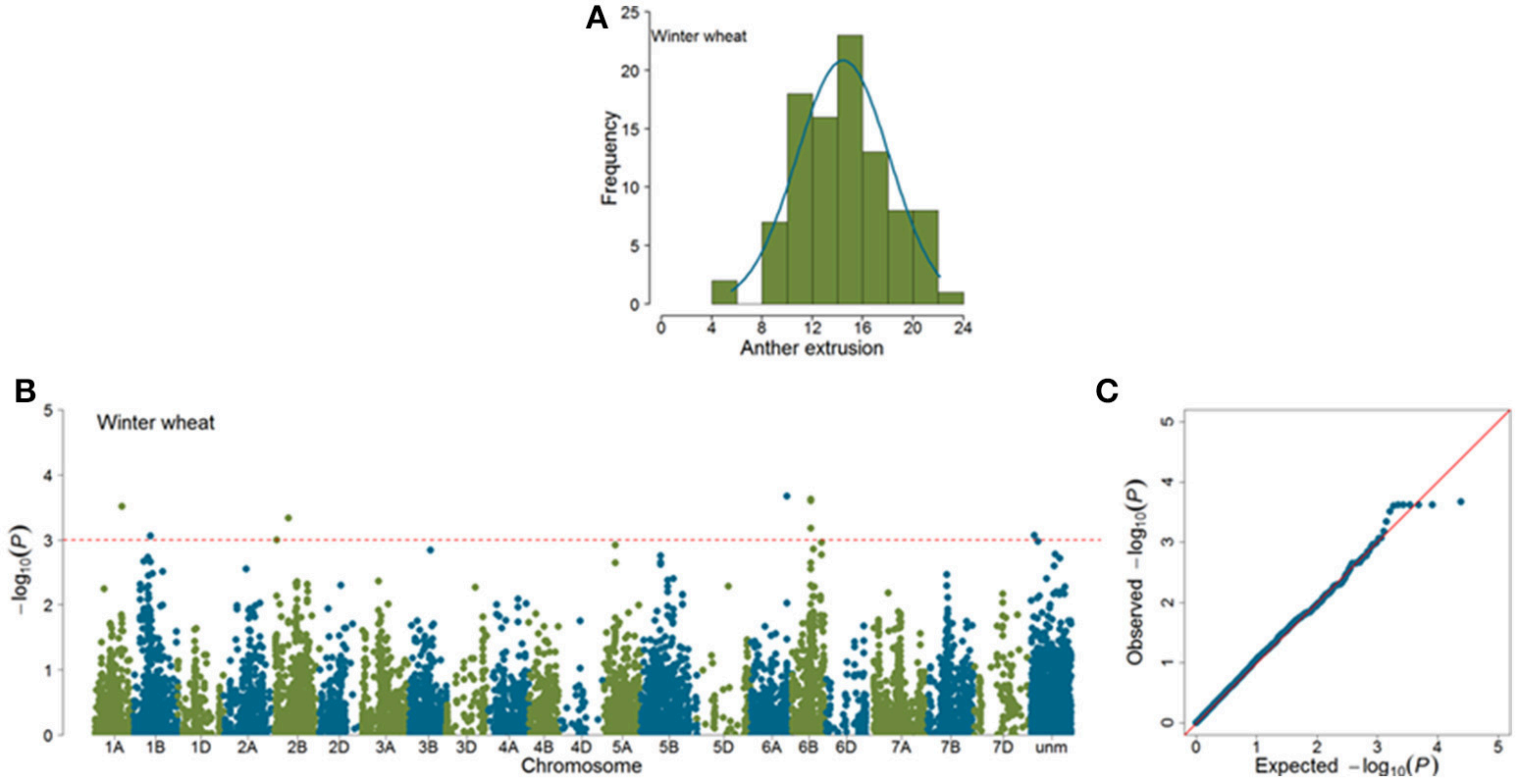
**Summary of genome-wide association studies results for anther extrusion (AE) in winter wheat panel (WP)**. **(A)** Distribution of Best Linear Unbiased Estimates of AE **(B)** Manhattan plots based on mixed linear model using kinship matrix (*K*) to correct for population stratification. Horizontal dashed line corresponds to the threshold (|*log*_10_(*P*)| > 3.0) for MTA estimation. **(C)** Quantile-quantile plots depicting expected vs. observed |*log*_10_(*P*)| values.

Marker-trait associations (MTAs) were identified for AE by using a mixed linear model (Yu et al., 2006) correcting for population stratification by using a kinship matrix (Lynch and Walsh, 1998) among pairs of accessions. In total, 23 (SP = 11; WP = 12) significant MTAs (|*log*_10_(*P*)| > 3.0) were identified in GWAS analyses (Figures 4, 5). Of the SP, nine significant markers had favorable additive effects, while two were unfavorable and the significant markers explained phenotypic variances (*R*^2^) ranging from 9.75 to 14.27%. The significant MTAs of SP were detected on chromosomes 2B, 3A, 3B, 6A, 7A, and 7B (Table 1 and Table S2). Of the WP significant markers, three markers had favorable while nine had unfavorable effects on AE and the markers explained phenotypic variances ranging from 9.44 to 16.98%. The WP significant MTAs were detected on chromosomes 1A, 1B, 2B, 6A, and 6B (Table 1 and Table S3). Chromosome 6A harbored MTAs in the genomic region (135–141 cM) shared by both panels. The quantile-quantile (qq) plots, which compare observed vs. expected |log_10_(*P*)| values, indicated that the model we used was sufficiently stringent to control for false positives (Figures 4, 5).

**Table 1:** **List of significant markers (−*log*_10_(*P*) > 3.0) for anther extrusion in spring and winter wheat panels based on their BLUEs values**.

**SNP**	**Panel**	**Chr**.	**Pos**.	**−log_10_(*P*)**	**Effect**	**%*R*^2^**	**Variant**	**Gene**
JD_c285_1438	Winter	1A	115.39	3.51	1.462	14.51	A	Traes_1AL_48F565722
Kukri_c51474_334	Winter	1B	70.08	3.06	−1.240	10.72	A	Traes_1BL_49BF78501
RAC875_c1755_971	Winter	2B	66.20	3.34	1.358	11.74	C	-
wsnp_Ex_c51352_55323092	Spring	2B	97.37	3.09	1.476	9.75	C	Traes_2BS_93E49A2FF
BS00013584_51	Spring	3A	77.51	3.14	1.519	11.35	A	Traes_3AS_491B176E2
GENE-4918_283	Spring	3B	57.24	3.92	2.003	13.83	T	TRAES3BF175400030CFD_g
BS00016407_51	Spring	3B	57.24	3.82	1.990	14.14	C	-
IAAV8162	Spring	3B	57.24	3.82	1.990	14.14	A	TRAES3BF175400030CFD_g
Excalibur_c25390_2483	Spring	6A	138.31	3.06	−1.421	10.93	A	Traes_6AL_65FBA1460
Excalibur_c3959_118	Winter	6A	140.87	3.67	−1.540	12.38	T	Traes_6AL_2A11939F7
wsnp_Ku_c16522_25425455	Winter	6B	71.18	3.18	−1.378	16.98	C	Traes_6BL_D816BA883
tplb0061f12_679	Winter	6B	71.76	3.62	−1.563	14.28	A	Traes_6BL_A7A807541
RFL_Contig1015_620	Winter	6B	71.76	3.62	−1.563	14.28	C	Traes_6BL_414885850
wsnp_Ku_c3185_5949143	Winter	6B	71.76	3.62	−1.563	14.28	T	Traes_6BL_9145A9A11
RAC875_rep_c108200_137	Winter	6B	71.76	3.62	−1.563	14.28	T	Traes_6BL_A5614E593
TA001470-0628	Winter	6B	71.76	3.62	−1.563	14.28	A	Traes_6BL_8960BC410
Kukri_rep_c116323_79	Winter	6B	71.76	3.60	−1.517	13.78	A	-
Tdurum_contig42487_1555	Spring	7A	212.66	4.36	−1.548	13.27	T	Traes_7AL_3ACEF0277
wsnp_BE443010B_Ta_2_1	Spring	7B	76.31	3.89	1.782	11.42	C	Traes_7BL_566C6F683
Excalibur_c20309_539	Spring	unm	unm	4.57	1.542	14.27	C	Traes_3DL_4A818FD98
Kukri_rep_c110544_248	Spring	unm	unm	4.33	1.507	14.24	A	Traes_3DL_C1FD5EB05
GENE-3863_132	Spring	unm	unm	3.16	1.548	10.14	G	Traes_6BL_729B4C623
wsnp_Ex_c3670_6694480	Winter	unm	unm	3.07	1.772	9.44	G	Traes_5AL_D05BE87FD

*unm stands for unmapped SNPs*.

*% R^2^ represents percentage of phenotypic variance imparted by individual SNP; Variant represents minor allele*.

### Study of genes in significant LD regions

Analysis of all the significant markers and the markers in significant LD regions (*r*^2^ ≥ 0.30) revealed 26 and 52 distinct markers in SP and WP, respectively. The corresponding orthologous genes/regions to these SNP markers were retrieved from wheat, barley, *Brachypodium distachyon*, sorghum and rice. Tables S2, S3 document the transcripts found in the different species for SP and WP, respectively.

**Figure 6:**
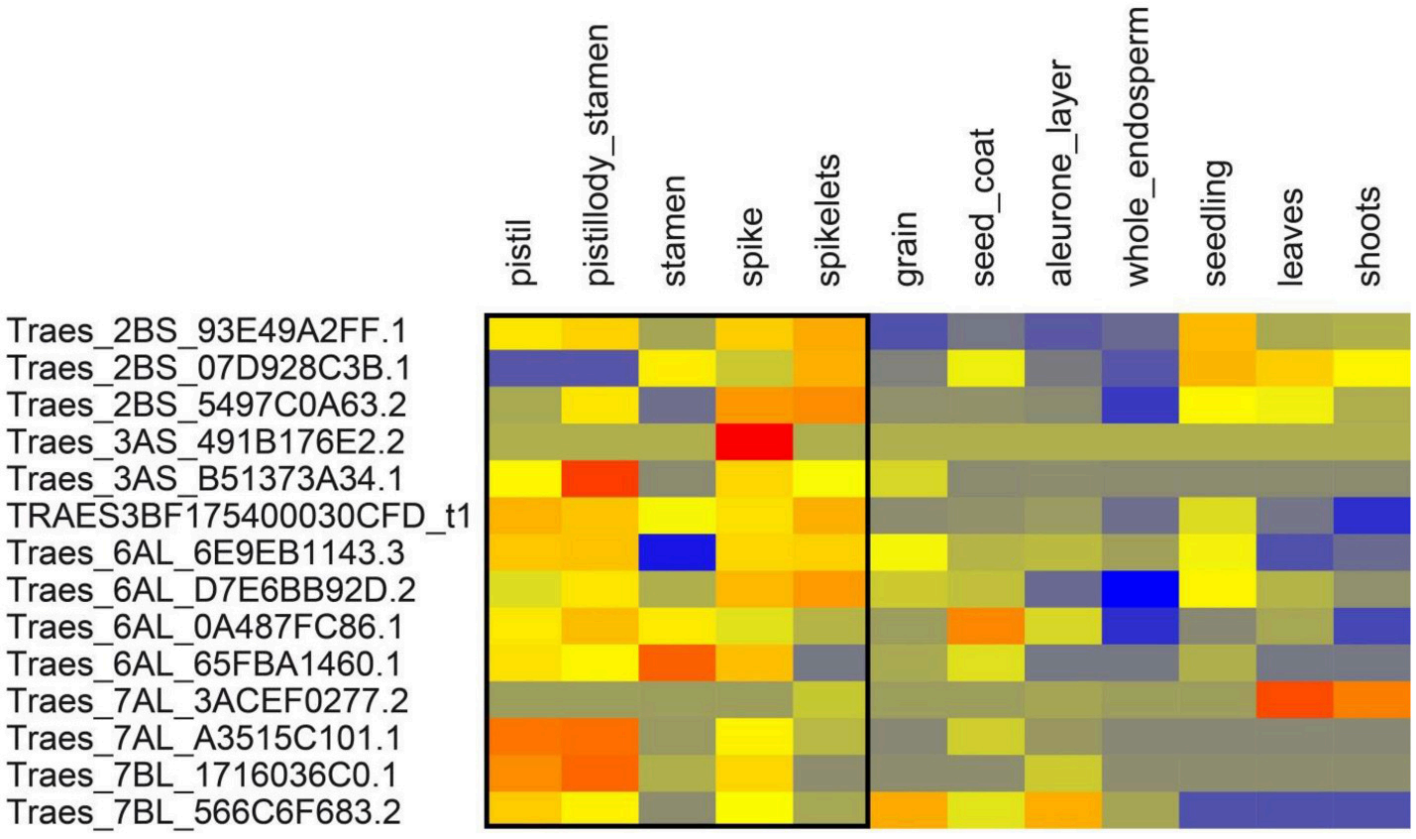
**Expression of genes from spring wheat panel in above ground tissues**. Expression values are given at log2 scale of transcripts per million (tpm): red, high expression; yellow, moderate expression; blue, low expression. Each individual gene (transcript_ID) is represented as horizontal row and different tissues are described in vertical columns. Tissues from pistil to spikelets show higher expression values for most genes compared to other tissues. Gene expression data were obtained from http://www.wheat-expression.com.

**Figure 7:**
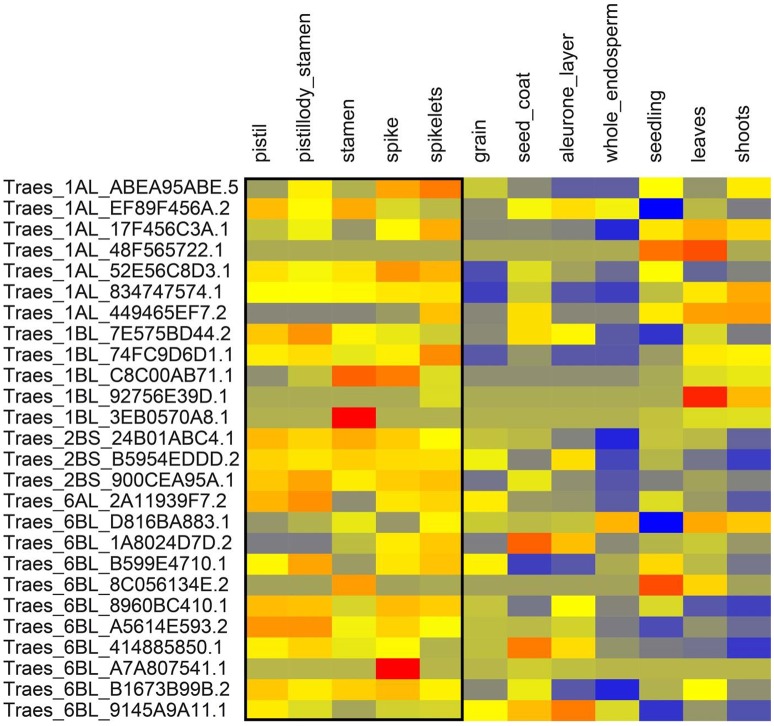
**Expression of genes from winter wheat panel in above ground tissues**. Expression values are given at log2 scale of transcripts per million (tpm): red, high expression; yellow, moderate expression; blue, low expression. Each individual gene (transcript_ID) is represented as horizontal row and different tissues are described in vertical columns. Tissues from pistil to spikelets show higher expression values for most genes compared to other tissues. Gene expression data were obtained from http://www.wheat-expression.com.

We extracted the wheat transcript IDs in significant LD regions and retrieved the gene expression data of above-ground tissues from http://www.wheat-expression.com. The expression data showed that some of these genes were expressed relatively higher in the tissues active at the anthesis stage i.e., pistils, stamens, spikes, and spikelets (Figures 6, 7). One way RM-ANOVA and all pair-wise multiple comparisons (Holm-Sidak method) on expression data (flowering vs. non-flowering tissues) suggested that the differences in the mean expression values between both groups are significant (*P* < 0.05) (Tables S4A,B). The genes and annotated functions of all the markers in significant LD region (*r*^2^ ≥ 0.30) revealed putative genes which could be directly or indirectly involved in AE in both panels.

## Discussion

### Anther extrusion is a heritable and phenotypically diverse trait

Anther extrusion (AE) was scored for a total of 207 wheat accessions including 111 spring (SP) and 96 winter (WP) over the period of 3 growing years which resulted in broad sense heritability values of 0.84 for SP and 0.65 for WP. Anther extrusion showed a strong phenotypic variation among accessions in both panels. The repeatability and broad sense heritability values for AE in both SP and WP complemented previous reports based on various wheat panels and different environments (Singh et al., 2007; Skinnes et al., 2010; Lu et al., 2013; Langer et al., 2014; Buerstmayr and Buerstmayr, 2015; Boeven et al., 2016). The continuous frequency distribution of AE suggested the possible contribution of several genes which was confirmed in the subsequent genetic analyses. These findings suggest that selection for AE could be performed to improve AE in wheat.

### SNP density and extent of LD are important factors for AE mapping

For efficient QTL detection, a high marker allele density increases the chances for the detection of linked QTL. We used 15K SNP array which guarantees the best polymorphic genetic markers taken from the 90K SNP chip reported by Wang et al. (2014). In our marker analysis, sub-genome-wise coverage was maximum for B-genome and least for D-genome in both spring and winter panels. A potential reason for the lowest marker coverage on D-genome is the lack of polymorphism reported for this genome (Akhunov et al., 2010). One reason may be the relative younger age (whereby less mutations could have accumulated) of the D-genome (ca. 1–2 million years) compared to A and B-genomes which diverged ca. 7 million years ago and which were potentially involved in its homoploid formation (Marcussen et al., 2014, but see also Li et al., 2015). Moreover, the D-genome hybrid origin itself would have been an intense bottleneck reducing genetic diversity. This least D-genome coverage is also seen by using other marker types, i.e., DArT and GBS markers (Muqaddasi et al., 2016, 2017). By using these resources, we detected several AE-QTL with moderate significances and effects. This result is in accordance with the studies about QTL mapping of AE (Skinnes et al., 2010; Lu et al., 2013; Buerstmayr and Buerstmayr, 2015; Muqaddasi et al., 2016). It implies that no single major gene is governing the floral architecture of wheat, but that the co-evolution of several genes resulted in the observed phenotypes. Nevertheless, one could imagine the pyramiding of several favorable AE-QTL in a variety.

Our linkage disequilibrium (LD) analysis resulted in extremely low *r*^2^ values between the adjacent pairs of marker loci. This suggested that due to the absence of closely linked SNPs especially on the D-genome, some QTLs may possibly have not been detected. As fast LD decay is a precondition for genome-wide association mapping, the increase of marker density may improve the detection of QTL for AE.

### Anther extrusion is controlled by concerted action of several genetic loci

Anther extrusion in our study behaved as a quantitative trait in both SP and WP; controlled by the concerted action of several loci and explaining a minor to modest level of phenotypic variance (*R*^2^(SP) = 9.75−14.24%; *R*^2^(WP) = 9.44−16.98%) for individual SNPs.

Our AM analysis revealed 11 significant SNPs in SP and 12 in WP, defining six (on chromosomes 2B, 3A, 3B, 6A, 7B, and 7D) and five (on chromosomes 1A, 1B, 2B, 6A, and 6B) QTL regions, respectively. Most studies published on AE are linkage-mapping studies (Skinnes et al., 2010; Lu et al., 2013; Buerstmayr and Buerstmayr, 2015) and used amplified fragment length polymorphism (AFLP), diversity array technology (DArT) and simple sequence repeat (SSR) makers. To our knowledge, AM studies published on the subject comprise of AM in spring wheat using mainly DArT and GBS markers (Muqaddasi et al., 2016, 2017) and SNP markers in winter wheat (Boeven et al., 2016). The use of dissimilar marker systems and/or background of mapping populations both in linkage and association mapping studies on AE make it challenging to compare the QTL intervals. However, comparison of QTL with previously published key QTL (imparting more than 10% of phenotypic variance) in the similar regions of chromosomes can give a better hint about the nature of AE and novelty of QTL.

For example, in our study chromosome 1A harbored a QTL (113.19–115.72 cM), represented by significant SNP “*JD_c285_1438*” in WP and explaining 14.51% of phenotypic variation (*R*^2^). Skinnes et al. (2010) detected a major QTL for AE on chromosome 1A in a bi-parental doubled haploid population which was located in the confidence interval between 92 and 102 cM (*R*^2^ = 18.3%). Similarly a WP-QTL on chromosome 6B (71.18–72.29 cM) imparted an average of 11.73% of phenotypic variation. Buerstmayr and Buerstmayr (2015) detected QTL for anther retention, an equivalent of AE, in a bi-parental mapping population on chromosome 6BL, with an interval covering a distance of 25 cM (~70–90 cM) and explaining a mean phenotypic variance of 11.3%. Based on the similar QTL regions in these two examples we assume they might constitute similar QTL, although the marker systems and populations are different. To our knowledge, no other AE-QTL explaining more than 10% of phenotypic variance has been reported in the similar regions to our QTLs and therefore these QTL can be considered novel.

Nevertheless, it is important to keep in mind that all recent reports suggest an extremely complex nature of AE which warrants that the AE is controlled by many loci, mostly small effect, contrary to what was initially reported that AE is controlled by very small number of genes (Sage and De Isturiz, 1974). Moreover, as most QTL are non-overlapping in earlier studies as well, it becomes apparent that AE greatly depends on the mapping population background and the marker system used. Moreover, our GWAS analyses yielded 70.76 and 63.94% of the total phenotypic variance imparted by SP and WP QTL, respectively. These results are analogous to all previous reports where a large part of the phenotypic variance remained unexplained which shows that marker density could be improved to capture the small effect loci and unexplained phenotypic variance.

### Synteny and expression data of significant LD regions reveal putative AE genes

We found several orthologous genes in close wheat relatives i.e., barley, *Brachypodium distachyon*, rice and sorghum. The annotated functions of several genes suggested that the proteins they encode can be important components of flowering and reproduction related traits, for example, D111/G-patch domain-containing protein (Quiapim et al., 2009), MADS-box (Theissen et al., 2000), nuclear pore anchor (Xu et al., 2007). Likewise, the expression profiles of most of these genes suggested a significantly higher expression in flowering tissues as compared to other non-flowering above-ground tissues. Based on annotated function and expression profiles, we suggest that these genes could either be the genes-of-interest or linked to the true gene-of-interest. However, further investigation and validation of the roles of these genes in wheat is still needed. These AE-associated genes can be considered as possible candidate genes and therefore provide a good resource for future map based cloning. Furthermore, additional genes in LD which were not present on the 15K SNP-chip may be the causal genes for AE-QTL.

## Conclusions

Hybrid wheat breeding is a promising strategy to break the yield barriers (Muhleisen et al., 2014; Zhao et al., 2015). Limited cross-pollination resulting from the cleistogamous nature of wheat is a major limitation to sufficient seed set for efficient hybrid production. High AE can ensure higher cross-fertilization rates for wheat hybrid formation. Our results show that AE is a stable trait, has high heritability rates and could be improved by breeding. Moreover, our GWAS results demonstrate that AE is a complex trait controlled by the combined action of several genetic loci. The favorable markers could be used to incorporate AE in high yielding varieties via marker assisted breeding to promote hybrid-heterosis in the key crop wheat.

## Author contributions

QM performed the data and genome-wide association analyses and prepared the manuscript. JB participated in data analysis. AB, KP, and MR participated in the experimental design. MR conceived the idea and participated in the interpretation of results and preparation of manuscript. All authors read and approved the final manuscript.

### Conflict of interest statement

The authors declare that the research was conducted in the absence of any commercial or financial relationships that could be construed as a potential conflict of interest.
