# Supplementary material for: Genetic Architecture of Anther Extrusion in Spring and Winter Wheat
Source: Front Plant Sci. 2017 May 16;8:754. doi: 10.3389/fpls.2017.00754 (PMC5432570; doi:10.3389/fpls.2017.00754)
Supplement: Supplementary file 5 [file DataSheet1.PDF]

*Supplementary Material*

# Genetic Architecture of Anther Extrusion in Spring and Winter Wheat

**Quddoos H. Muqaddasi*, Jonathan Brassac, Andreas Börner, Klaus Pillen and Marion S. Röder**

***Correspondence:** muqaddasi@ipk-gatersleben.de

## Supplementary Figures

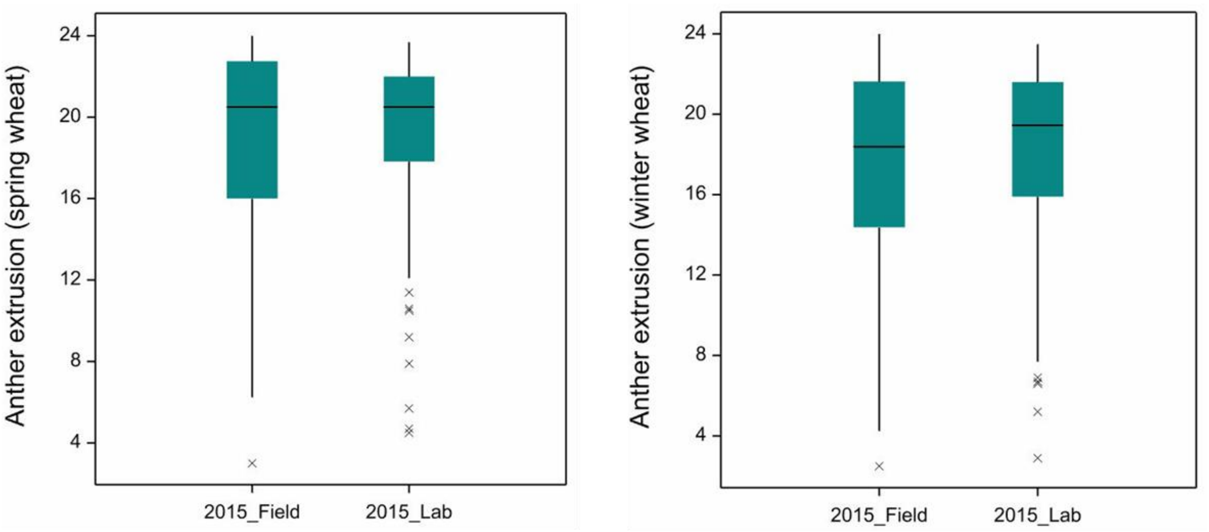


***r* = 0.81, *P* <0.0001**  ***r* = 0.85, *P* <0.0001**

**Figure S1.** Box plots for field and lab based methods for scoring of anther extrusion in spring and winter wheat panels. Pearson's correlation (*r*) and significance of genetic variation (*P*) is given under the figure.

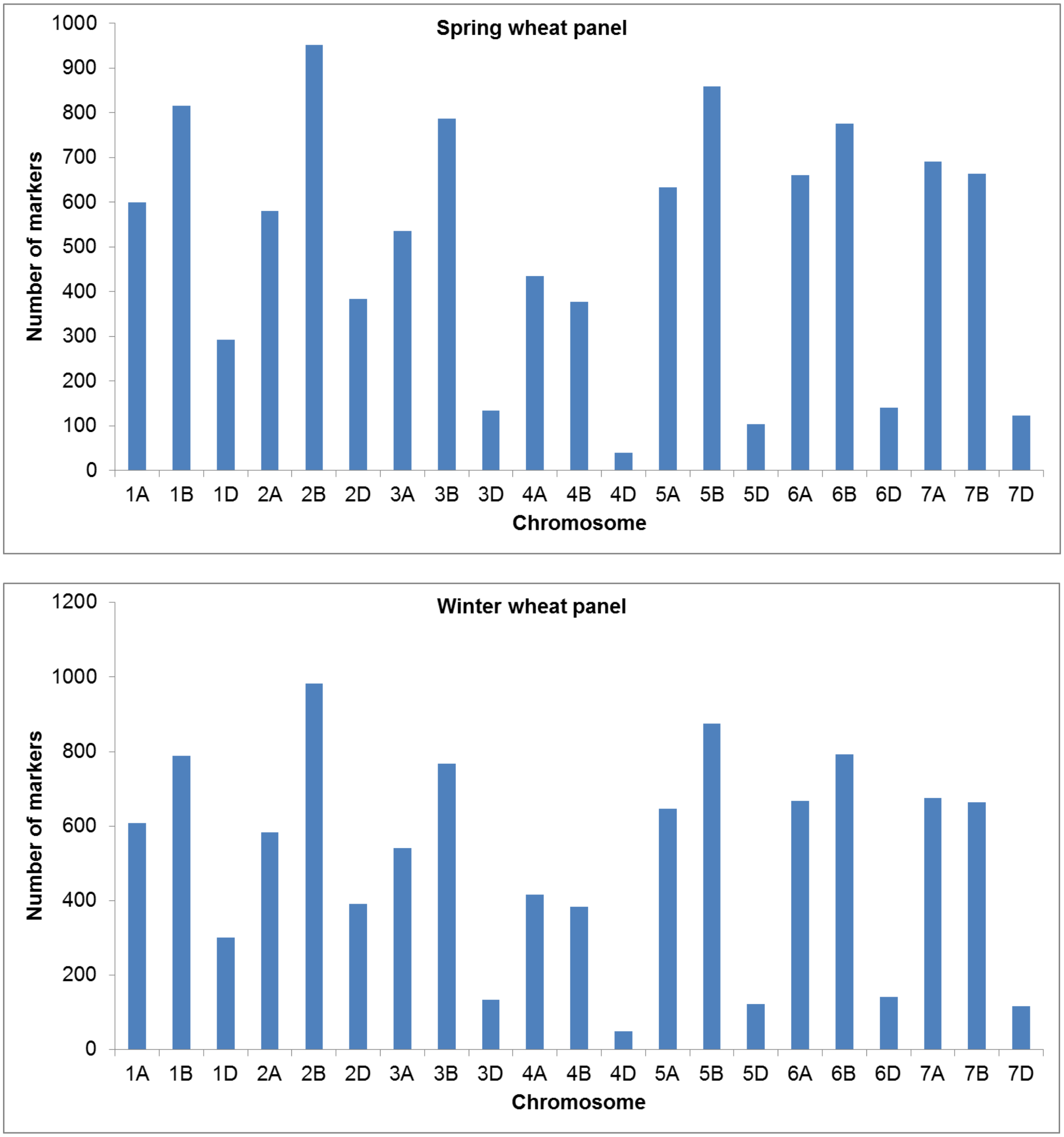


**Figure S2.** The chromosome-by-chromosome distribution of the set of mapped SNP markers in spring and winter wheat panels.

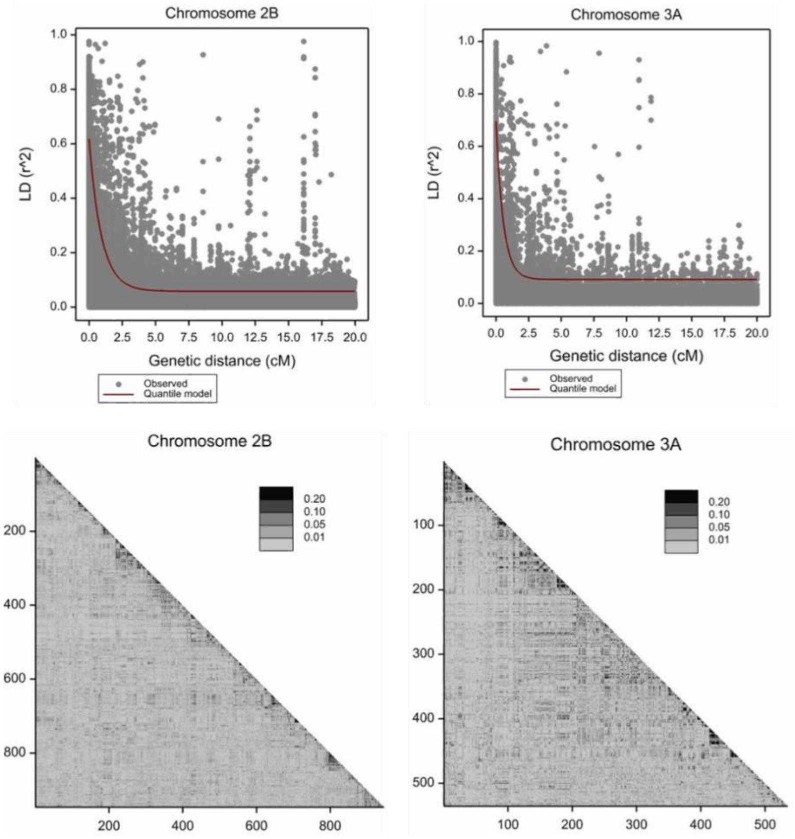
Chromosome 2B
LD (r^2)
Genetic distance (cM)
Observed
Quantile model
Chromosome 3A
LD (r^2)
Genetic distance (cM)
Observed
Quantile model
Chromosome 2B
0.20
0.10
0.05
0.01
Chromosome 3A
0.20
0.10
0.05
0.01

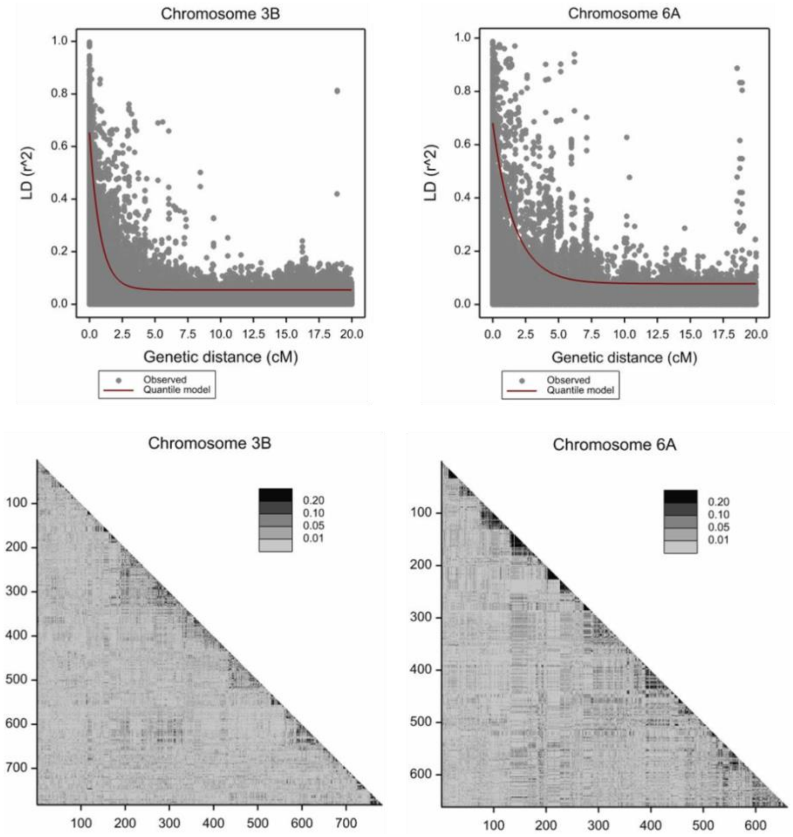
Chromosome 3B
LD (r^2)
Genetic distance (cM)
Observed
Quantile model
Chromosome 6A
LD (r^2)
Genetic distance (cM)
Observed
Quantile model
Chromosome 3B
0.20
0.10
0.05
0.01
Chromosome 6A
0.20
0.10
0.05
0.01

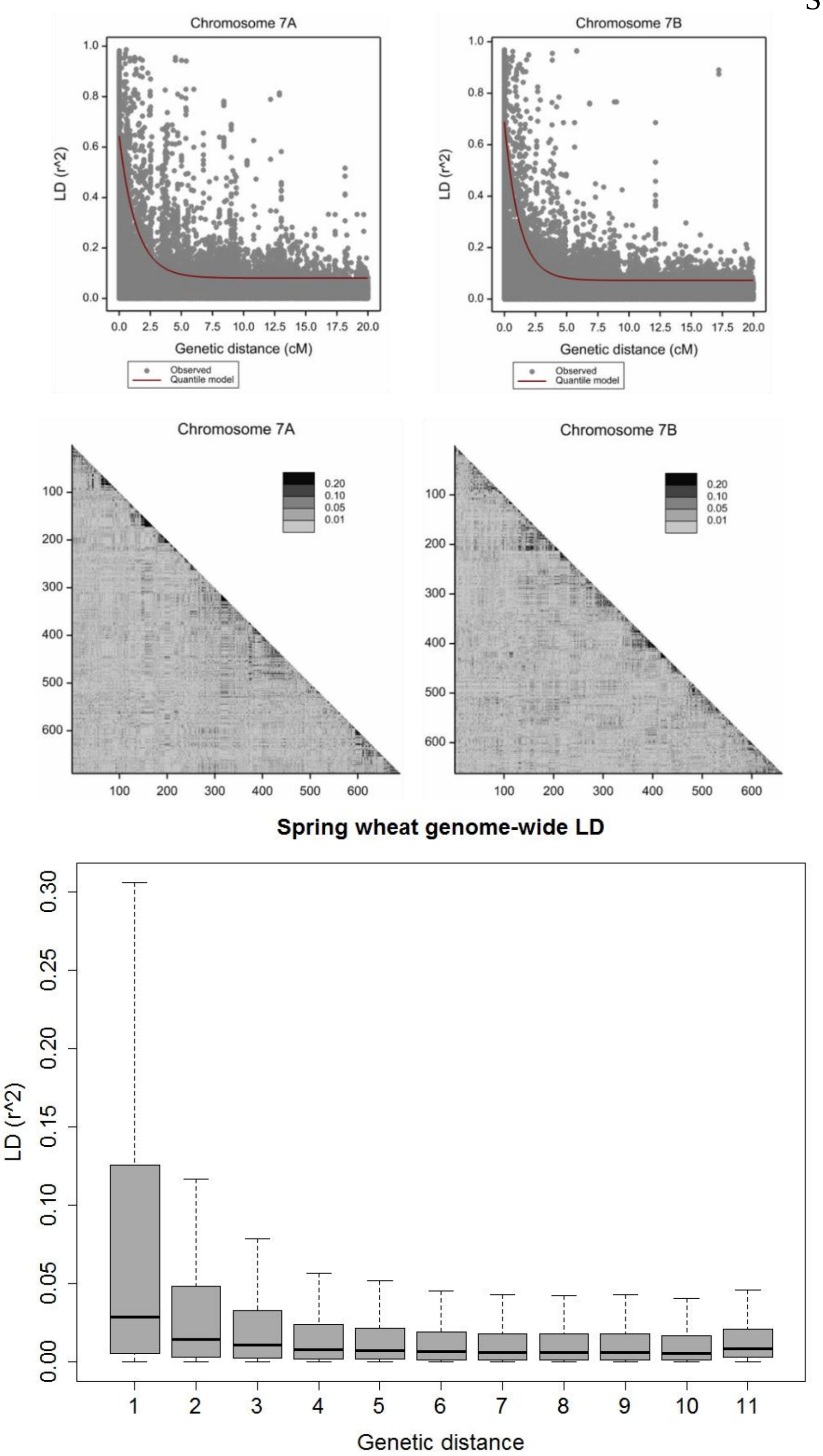


**Figure S3.** Linkage disequilibrium (LD) plots in spring wheat panel. All representative chromosomes harbor the significant markers ($|\log_{10}(P)|$ >3.0) associated with anther extrusion. Heat maps depict $r^2$ between the marker pairs. Intra-chromosomal and genome-wide plots of LD decay show $r^2$ against the genetic distances (cM) between pairs of SNP loci.

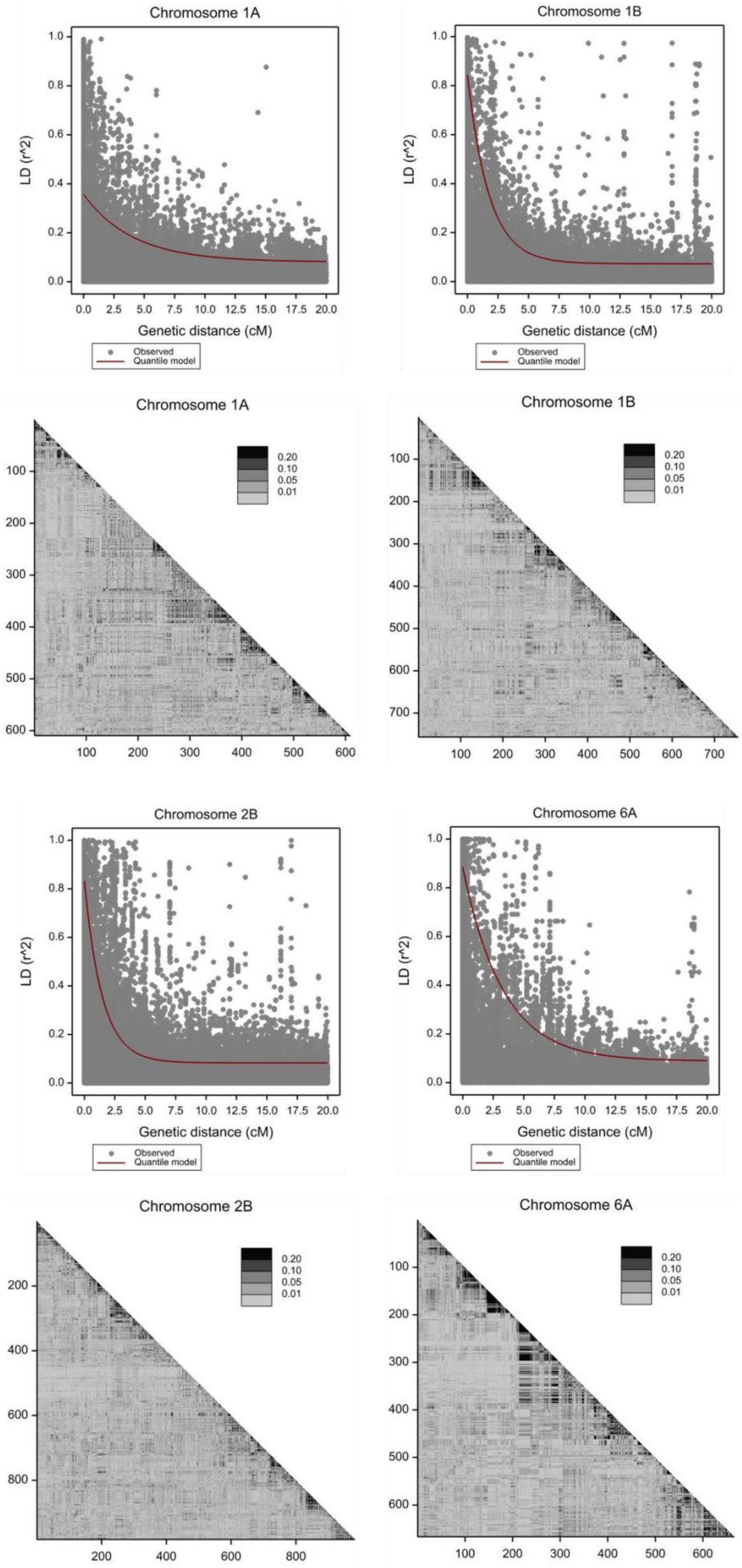
Chromosome 1A
LD (r^2)
Genetic distance (cM)
Observed
Quantile model
Chromosome 1B
LD (r^2)
Genetic distance (cM)
Observed
Quantile model
Chromosome 1A
0.20
0.10
0.05
0.01
Chromosome 1B
0.20
0.10
0.05
0.01
Chromosome 2B
LD (r^2)
Genetic distance (cM)
Observed
Quantile model
Chromosome 6A
LD (r^2)
Genetic distance (cM)
Observed
Quantile model
Chromosome 2B
0.20
0.10
0.05
0.01
Chromosome 6A
0.20
0.10
0.05
0.01

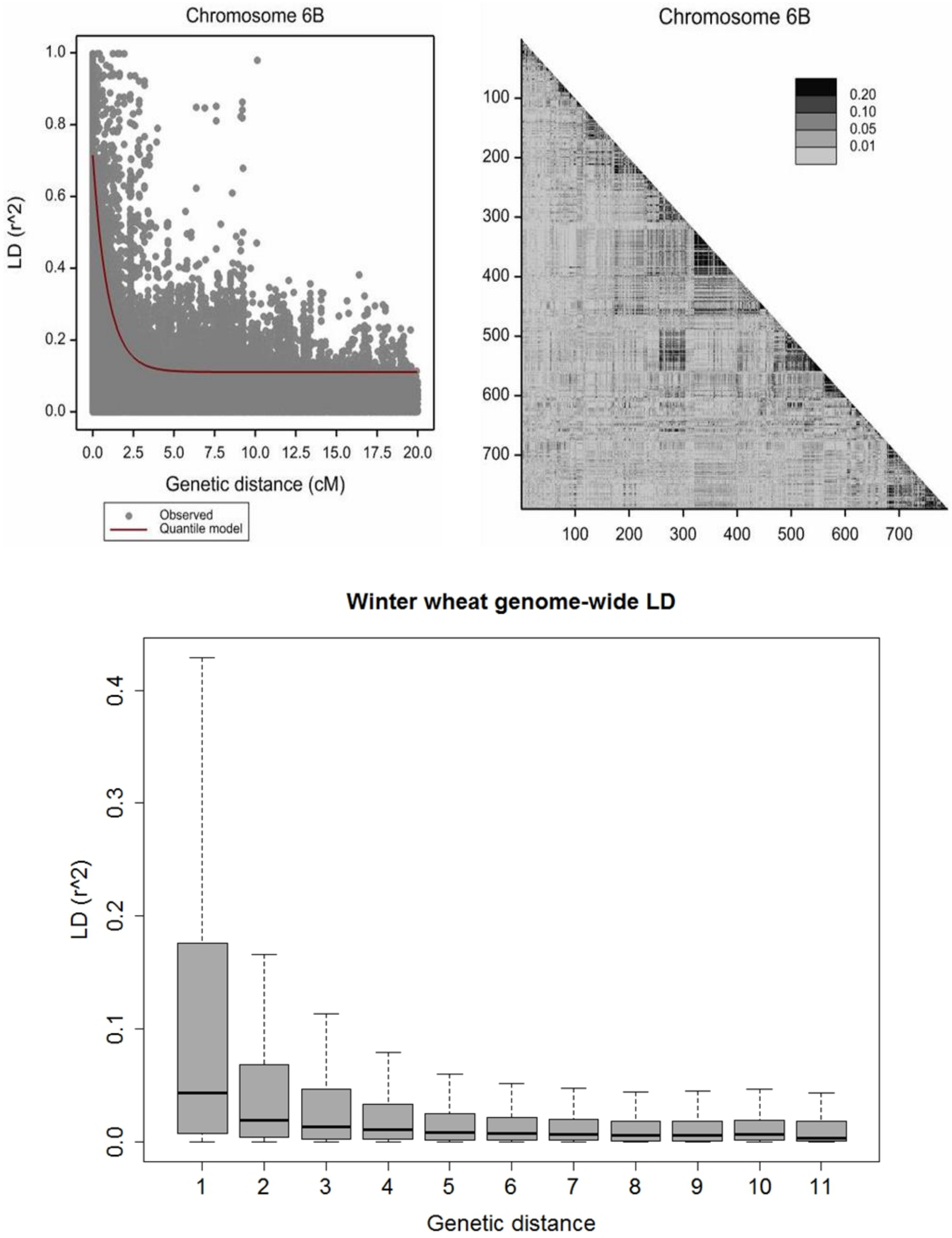


**Figure S4.** Linkage disequilibrium (LD) plots in winter wheat panel. All representative chromosomes harbor the significant markers ($|\log_{10}(P)| > 3.0$) associated with anther extrusion. Heat maps depict $r^2$ between the marker pairs. Intra-chromosomal and genome-wide plots of LD decay show $r^2$ against the genetic distances (cM) between pairs of SNP loci.
